# Is clinical effect of autologous conditioned serum in spontaneously occurring equine articular lameness related to ACS cytokine profile?

**DOI:** 10.1186/s12917-020-02391-7

**Published:** 2020-06-08

**Authors:** Patrick Marques-Smith, Anne S. Kallerud, Grethe M. Johansen, Preben Boysen, Anna M. Jacobsen, Karoline M. Reitan, Mia M. Henriksen, Maria Löfgren, Cathrine T. Fjordbakk

**Affiliations:** 1 Department of Companion Animal Clinical Sciences, Faculty of Veterinary Medicine, Norwegian University of Life Sciences, 0102 Oslo, Norway; 2 Department of Food Safety and Infection Biology, Faculty of Veterinary Medicine, Norwegian University of Life Sciences, 0102 Oslo, Norway; 3 Department of Biomedical Science and Veterinary Public Health, Swedish University of Agricultural Sciences, 75007 Uppsala, Sweden

**Keywords:** Biologic therapies, Cytokines, Growth factors, Intra-articular treatment

## Abstract

**Background:**

Biologic’ therapies, such as autologous conditioned serum (ACS), are gaining popularity in treating orthopaedic conditions in equine veterinary medicine. Evidence is scarce regarding ACS constituents, and large inter-individual differences in cytokine and growth factor content have been demonstrated. The objective of the current study was to investigate the potential association between cytokine and growth factor content of ACS and clinical effect in harness racehorses with spontaneously occurring low-grade articular lameness. Horses received 3 intra-articular injections of ACS administered at approximately 2-week intervals. Lameness evaluation consisting of a trot-up with subsequent flexions tests was performed at inclusion and approximately 2 weeks after the last treatment (re-evaluation); horses were classified as responders when there was no detectable lameness on trot-up and a minimum of 50% reduction in flexion test scores at re-evaluation. Association between clinical outcome (responders vs. non-responders) and age, lameness grades at inclusion (both initial trot-up and after flexion tests), treatment interval, follow-up time and the ACS content of IL-1Ra, IGF-1 and TGF-β was determined by regression modelling.

**Results:**

Outcome analysis was available for 19 of 20 included horses; 11 responded to treatment whereas 8 did not. There was considerable inter-individual variability in cytokine/growth factor content of ACS, and in the majority of the horses, the level of IL-10, IL-1β and TNF-α was below the detection limit. In the final multivariate logistic regression model, ACS content of IGF-1 and IL-1Ra was significantly associated with clinical response (*P* = 0.01 and *P* = 0.03, respectively). No association with clinical response was found for the other tested variables.

**Conclusions:**

The therapeutic benefit of ACS may be related to higher levels of IL-1Ra and IGF-1. Our study corroborates previous findings of considerable inter-individual variability of cytokine- and growth factor content in ACS.

## Background

Since the report of its effectiveness in improving lameness in horses with experimentally induced carpal osteoarthritis (OA), intra-articular treatment with autologous conditioned serum (ACS) is becoming increasingly more popular in equine practice [1, 2]. ACS is a biologic blood product obtained by aseptic incubation of the patient’s blood in the presence of monocyte-activating surfaces, followed by centrifugation and subsequent extraction of the serum fraction [3, 4]. Compared to un-manipulated blood, ACS contains increased amounts of anti-inflammatory cytokines and growth factors such as interleukin 1 receptor antagonist (IL-1Ra), interleukin 10 (IL-10), transforming growth factor β (TGF-β), insulin-like growth factor 1 (IGF-1) and platelet-derived growth factor (PDGF) [3–7]. Although the exact composition of ACS is unknown, the proposed disease-modifying mechanism in joint disease is believed to be due to IL-1Ra blocking the IL-1 receptor, thereby preventing detrimental effects of IL-1β on articular tissues in OA pathophysiology [3, 8, 9]. However, for effective receptor blockade to occur, IL-1Ra has to significantly out-number IL-1β; in human chondrocytes, the required ratio for 50% inhibition of IL-1 activity has been reported as approximately 100:1 [10]. In horses, the optimal therapeutic IL-1Ra:IL-1β ratio has not been determined [11].

Considerable inter-individual differences in ACS constituents have been reported by several research groups [4, 12, 13], and current literature has failed to identify a preparation method where such variability is limited or negligible [4, 7, 12, 13]. Although patient-related factors such as inflammatory status have been shown to influence ACS cytokine profile [12], predictors for cytokine and growth factor production in ACS have yet to be identified. Consequently, if the therapeutic effect of ACS is determined by the levels of the anti-inflammatory constituents known to date, using ACS in equine clinical practice could be considered a hit-and-miss approach, as there is no method of identifying animals with sub-therapeutic ACS cytokine levels. On the other hand, short clearance time of ACS cytokines in vivo supports the counter argument that cytokine profile of ACS is of little therapeutic significance [6]. Therefore, as a first step in investigating a potential link between cytokine profile and therapeutic efficacy of ACS, the objective of the current study was to evaluate clinical response to ACS in horses with spontaneously occurring low-grade articular lameness, and to investigate the potential association between cytokine and growth factor content of ACS and treatment response. Based on previous clinical experience, we hypothesized that treated horses would fall into one of two outcome categories (responders and non-responders), and that these categories would be associated with the cytokine and growth factor profile of the ACS.

## Results

### Case details and clinical examination

Included horses comprised 12 Norwegian-Swedish Coldblooded trotters and 8 Standardbreds; see Table 1 for case details. Subjective and objective lameness evaluation including diagnostic anaesthesia was performed as described in the methods section. Ten horses presented with lameness localized to one joint in one limb; one horse presented with lameness localized to two joints in one limb. Two-limb lameness was identified in 9 horses; among these, 5 horses presented with bilateral forelimb lameness in which lameness of the least lame limb (secondary problem) could only be detected after successfully alleviating the lameness of the most lame limb (primary problem) with diagnostic anaesthesia. Three horses had ipsilateral fore and hind limb lameness, whereas one horse presented with diagonal fore and hind limb lameness (Table 1). In all horses, a primary problem as defined in the methods section could be identified; the primary problem affected a forelimb in 14 horses and a hind limb in 6 horses (Table 1).

**Table 1:** Study population

Case no.	Breed	Age	Sex	1° problem	2° problem	Radiographic findings	Responder
**1**	NSCT	5	G	R carpus	–	WNL	Y
**2**	NSCT	2	G	L carpus	–	Palmar deviation of the carpus (calf kneed)	N
**3**	STB	6	F	L carpus	–	Mild heterogenous radiopacity RCB	Y
**4**	NSCT	4	M	L TCJ	RF MCPJ	WNL	Y
**5**	STB	2	F	L carpus	–	Smoothly marginated non-union fracture proximal MCII	N
**6**	STB	3	G	L MCPJ	R MCPJ	Mild remodeling dorsoproximal medial P1, L	Y
**7**	NSCT	2	F	L carpus	–	Mild remodeling dorsoproximal RCB	Y
**8**	NSCT	7	F	L carpus	L MCPJ	Mild remodeling dorsodistal medial radius and dorsoproximal RCB	N
**9**	NSCT	5	F	L TCJ	L carpus	Mild remodeling dorsodistal RCB	N
**10**	NSCT	3	G	L MCPJ	R MCPJ	Mild remodeling dorsoproximal medial P1, bilateral	N
**11**	NSCT	7	F	LF DIPJ	R DIPJ	WNL	Y
**12**	STB	4	G	L MCPJ	R MCPJ	Mild remodeling dorsoproximal medial P1, bilateral. Mild remodeling palmarodistal medial PSB, bilateral	Y
**13**	NSCT	7	G	R stifle	R MCPJ	WNL	NA
**14**	NSCT	5	G	R MCPJ	R MTPJ	WNL	Y
**15**	STB	6	G	L MCPJ	R MCPJ	Mild remodeling dorsoproximal medial P1, bilateral	Y
**16**	NSCT	5	F	L TCJ	–	Mild heterogeneous radiopacity of the medial malleolus of the tibia	N
**17**	STB	7	F	R MTPJ	–	WNL	N
**18**	STB	7	G	L MCPJ	–	Mild remodeling dorsoproximal medial P1 with a small dorsomedial osteochondral fragment	N
**19**	NSCT	4	G	R TCJ	–	WNL	N
**20**	STB	5	G	LF DIPJ	–	WNL	Y

Case details including radiographic findings and treatment outcome (responder Y: yes, N: no, NA: not available) for horses included in the study. *NSCT* Norwegian-Swedish Coldblooded Trotter, *STB* Standardbred, *G* gelding, *F* female, *M* intact male, *R* right, *L* left, *LF* left front, *RF* right front, *TCJ* tarsocrural joint, *MCPJ* metacarpophalangeal joint, *DIPJ* distal interphalangeal joint, *RCB* radiocarpal bone, *MCII* second metacarpal bone, *P1* proximal phalanx, *PSB* proximal sesamoid bone, *WNL* within normal limit.

When assessing the most lame limb at the initial trot-up, mean AAEP (American Association of Equine Practitioners) score was 1/5 (median 1/5) and the mean objective lameness measurements were 16.1 ± 10.8 mm for forelimbs and 5.9 ± 2.9 mm for hind limbs, respectively. When assessing the most severe flexion test at inclusion, mean AAEP score was 2/5 (median 2/5) and the mean objective lameness measurements were 37.9 ± 15.0 mm for forelimbs and 18.6 ± 4.1 mm for hind limbs, respectively. Radiographic findings are presented in Table 1.

### Treatment and re-evaluation

Horses were treated with 3 ACS injections, mean treatment interval was 14 days (median 15 days). Adverse effects were noted for one horse (Case 13) in which the third ACS injection of the medial femorotibial joint resulted in septic arthritis; this horse was subsequently excluded from re-evaluation and outcome analysis.

For all horses, mean time from inclusion to re-evaluation was 48 days (median 43 days), and the mean AAEP score at re-evaluation trot-up was 0.5/5 (median 0/5); the mean objective lameness measurements were 6.9 ± 4.4 mm for forelimbs and 7.8 ± 2.6 mm for hind limbs, respectively; mean AAEP score after flexion was 0.5/5 (median 1/5), and the mean objective lameness measurements after flexion were 15.0 ± 7.5 mm for forelimbs and 10.5 ± 5.8 mm for hind limbs, respectively. At re-evaluation, joint effusion was subjectively improved in 8 horses (6 responders and 2 non-responders); unchanged in 10 (5 responders and 5 non-responders), and more pronounced in 1 horse (non-responder).

### Outcome analyses

**Fig. 1:**
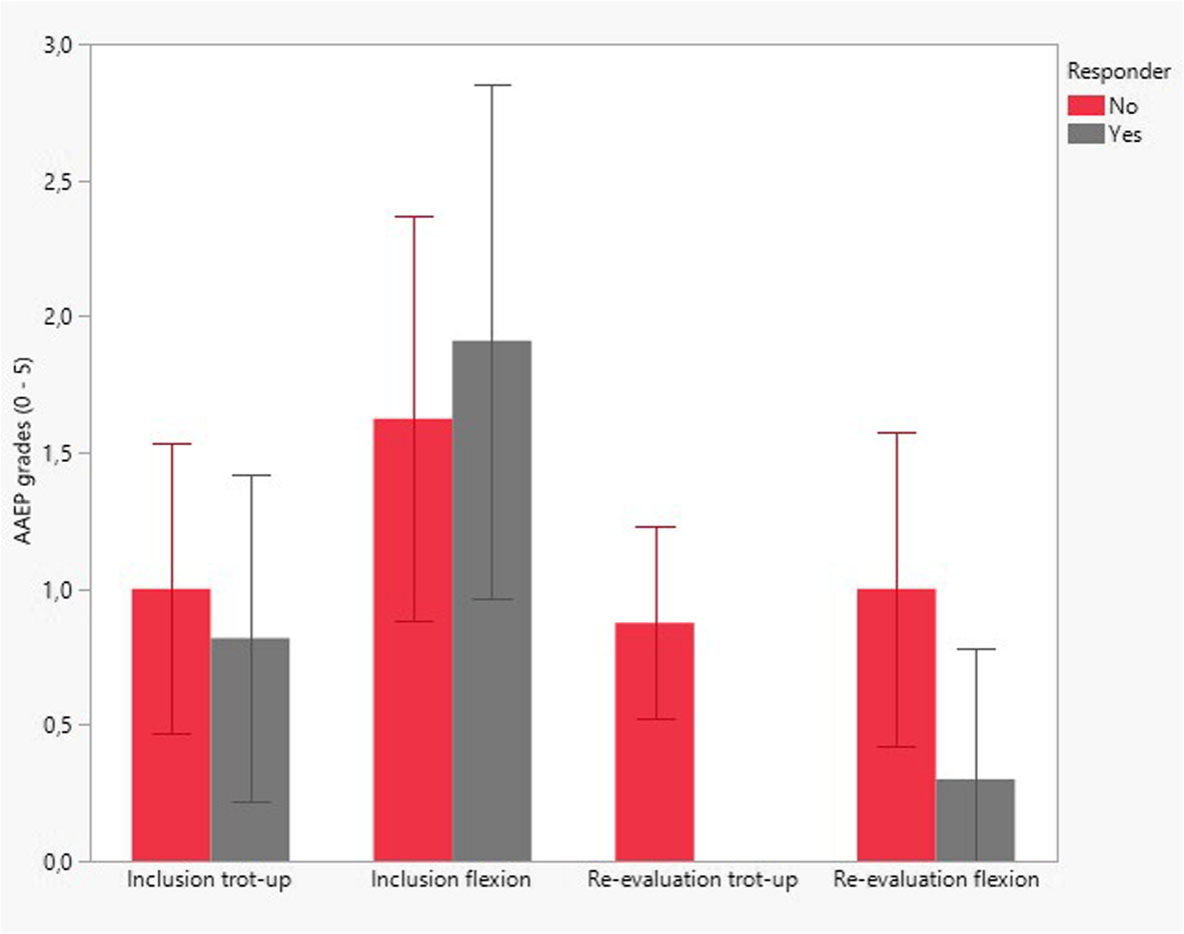
Bar graphs (mean ± standard deviation) illustrating lameness grades (AAEP scale; 0–5) at inclusion (trot-up and flexion) and at re-evaluation (trot-up and flexion) in 19 horses included in the study for which outcome data was available. Re-evaluation grades were used to categorize horses into responders (grey bars; *n* = 11) and non-responders (red bars, *n* = 8) to treatment. There was no statistical difference in inclusion grades between the outcome groups

The objective measurements were significantly correlated to the AAEP scores (for forelimb assessments, correlation coefficient was 0.82, *P* < 0.0001; whereas for hind limb assessments, correlation coefficient was 0.79, *P* = 0.0071); therefore, only the AAEP scores were used for further analyses. Based on the pre-set criteria defined in the methods section, 11 horses were classified as responders to treatment, whereas 8 horses were defined as non-responders (Fig. 1). When comparing these two groups, there were no statistically significant differences in lameness scores at inclusion (mean and median AAEP trot-up score 1/5 in both groups, respectively, *P* = 0.51; mean and median AAEP flexion test score 2/5 in the responders and 1.5/5 in the non-responders, respectively, *P* = 0.52). There were no statistically significant differences between the outcome groups in age (5.1 ± 1.5 years in responders, and 4.9 ± 1.5 years in non-responders, respectively, *P* = 0.77); treatment interval (16.8 ± 4.1 days in responders and 16.1 ± 3.4 days in non-responders, respectively, *P* = 0.56); follow-up time (48 ± 16 days for responders, and 43 ± 2 days for non-responders respectively, *P* = 0.4); or ACS volume used (13.1 ± 4 ml in responders and 14.1 ± 2.7 ml in the non-responders, respectively; *P* = 0.56).

**Fig. 2:**
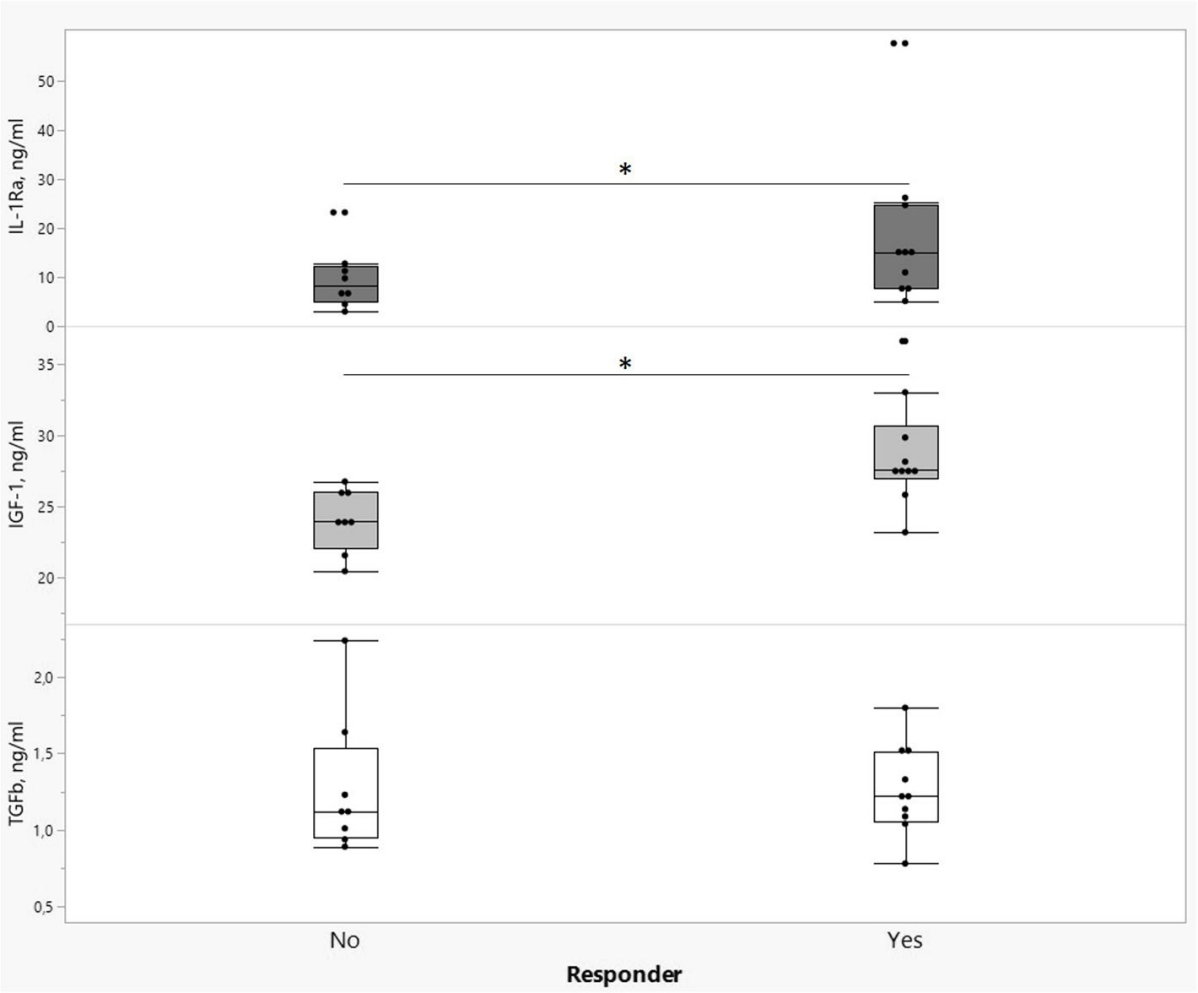
Box-and-whisker plots illustrating ACS content (ng/ml) of IL-1Ra (dark grey boxes), IGF-1 (light grey boxes) and TGF-β (white boxes) in horses categorized as responders (*n =* 11) versus non-responders (*n* = 8) to ACS treatment. Asterisks indicate significant differences between outcome groups (*P < 0.05*)

The ACS content of IL-1Ra, IGF-1 and TGF-β is detailed in Fig. 2. The majority of IL-10, IL-1β and TNF-α measurements fell below or near the lower limit of detection even at the lowest possible dilution and were therefore omitted from statistical analyses (data not shown).

Through univariate logistic regression modelling, a significant association between the outcome groups and the ACS levels of IGF-1 (*P* = 0.014) and IL-1Ra (*P* = 0.04) was identified (Fig. 2). No association between outcome and lameness scores at inclusion (initial trot-up and flexion tests, *P* = 0.66 and *P* = 0.61, respectively), age (*P* = 0.98), treatment interval (*P* = 0.66), time to follow-up (*P* = 0.15); volume of ACS used (*P* = 0.53) or the ACS levels of TGF- β (*P* = 0.63) was found. When including ACS levels of IGF-1 and IL-1Ra in the multivariate regression model, both factors were significantly associated with outcome (*P* = 0.01 and *P* = 0.04, respectively), with higher levels found in horses that responded to treatment (Fig. 2).

## Discussion

Results from the current study demonstrate that 58% of included horses responded to ACS treatment, and that these horses had higher ACS levels of IL-1Ra and IGF-1 than non-responders. The other investigated ACS constituents were either not found statistically different (TGF-β) or were at too low levels to be assessed (IL-1β, IL-10 and TNF-α). To the authors’ knowledge, this is the first study relating ACS content of IL-1Ra and IGF-1 to treatment outcome in spontaneously occurring low grade equine articular lameness.

Albeit small, the treatment effect reported in the current study is corroborated by clinical studies in human patients with knee OA [14, 15] as well as in an experimental model of induced equine carpal OA [1]. However, when evaluating the effect of ACS in vitro*,* only minimal beneficial effects on equine chondrocyte metabolism were found [16]. Discrepancies in reported ACS effects should be interpreted in the light of the different models used, where in vitro models might be too simplistic for replicating the complex pathophysiology taking place in a diseased synovial joint. On the other hand, the smaller clinical response observed in our study compared to the experimental study by Frisbie et al. [1], could potentially be due to the heterogenous nature of joint pathology and disease duration characterizing spontaneous traumatic OA [17]. In order to limit this variability, our study population consisted of horses with low grade lameness only. However, as clinical signs including lameness grade and absence of radiographic abnormalities may correlate poorly with the severity of the underlying joint disease [17], the non-responders in our study might have been affected by more severe joint disease including subchondral bone pathology, than the responders. Advanced diagnostics such as magnetic resonance imaging or computed tomography could have revealed underlying pathology especially of the subchondral bone [18] whereas articular cartilage health could have been evaluated by diagnostic arthroscopy. However, the substantial cost and relative invasiveness of such procedures negated their routine use in the current study.

The association between treatment response and ACS levels of IL-1Ra and IGF-1 was not surprising, as previous studies have demonstrated positive effects of both of these mediators on joint health [9, 19, 20]. The anti-inflammatory/anti-catabolic effect of IL-1Ra is dose-dependent, as an excess of IL-1Ra relative to IL-1 is necessary for functional IL-1 receptor blockade to occur [21]. The anabolic effects of IGF-1, such as stimulation of matrix aggrecan and collagen synthesis, are also dose-dependent [22]. Therefore, the contention that higher IL-1Ra and IGF-1 ACS levels would positively influence treatment response, seems logical. In fact, delivering a combination of these two mediators by gene therapy has been studied in vitro [23] as well as in vivo [24]. An additive effect on repair processes in chondrocytes depleted by IL-1β was seen when using IL-1Ra in combination with IGF-1 versus IGF-1 alone [23]. When treating experimentally created cartilage defects in equine joints, the IL-1Ra/IGF-1 combination resulted in improved quality of the repair tissue compared to placebo [24].

The treatment protocol used in the current study was based on the commonly used clinical protocol of 3 injections spaced approximately 2 weeks apart; this protocol is, however, not founded on scientific evidence but rather on clinical opinion and experience. Recently, a 2-day injection interval was found superior to the traditional weekly regime in reducing synovial fluid biomarkers of joint inflammation in horses diagnosed with OA over a follow-up period of 42 days; however, clinical effects were not reported [13]. If clinically effective, this treatment protocol would be of interest to the equine industry, as a shorter treatment duration could potentially lead to fewer days away from training. The long treatment duration was particularly problematic to owners and trainers of non-responders in the current study and both from a veterinary as well as an industry perspective, identifying potential non-responders prior to initiation of ACS therapy would have been ideal. Previous attempts at predicting ACS profile based on patient factors such as leukocyte levels have failed [12], most likely due to the large inter-individual variations in ACS cytokine levels. In humans, part of this variation is explained by genetic polymorphism, where monocyte production of IL-1Ra and IL-1β is dictated by the alleles at the IL-1Ra gene [25]. While genetic polymorphism at this gene might be present in horses as well, other factors such as inflammatory status also influence ACS profile [12]. Thus, if the clinical response associated to ACS content indicated by our data is corroborated by larger studies, having a stall-side kit for analysis of ACS constituents such as IL-1Ra could potentially be used to guide patient selection for similar treatments in the future.

Adverse effects of treatment with ACS were identified in one horse in the current study, which developed septic arthritis of the medial femorotibial joint after receiving the third ACS injection. This reaction was attributed to the procedure rather than the ACS itself, as the reaction affected only one of several injected joint compartments. In general, the risk of adverse effects of intra-articular ACS treatment is low, and in humans comparable to injections of saline [15].

The main limitation of our study was the small sample size. As such, it is possible that additional associations between clinical outcome and ACS constituents could have been detected if investigated in a larger population. Also, the inclusion of different limbs and joints added to the clinical heterogeneity of our material, and finally, although we aimed for standardized treatment and re-evaluation intervals, owner availability and convenience resulted in some variability for these parameters. Having the horses stabled at the institution for the duration of the study would have negated this problem; this was however cost prohibitive for the current study. However, no significant differences in treatment interval and time to re-evaluation were found between groups and neither were these parameters correlated with response to treatment.

During study planning, inclusion of a control group was considered. However, due to the studied population being client-owned horses and the negative impact such design would have on the client’s willingness to participate and remain in the study, this alternative was not considered feasible. Also, the rationale for treating all joints identified as a source of pain was based on ethical considerations (i.e refraining from treating a diagnosed joint problem), as well as uncertainty regarding client compliance where any perceived delay in treatment and return to training would strongly discourage study participation. The decision to restrict analysis of treatment response to the primary problem also has some limitations. While not ideal (optimally, a more homogenous population with a single affected joint would be preferred) we believe it represents the best choice of methodology in the context of this study. We were concerned about overestimating response to flexion tests when using the objective system, which was why subjective lameness scores were used instead, when evaluating response to treatment. The use of flexion tests in lameness evaluation is controversial as opinions differ regarding their value. However, the use of flexion tests is still common practice among racehorse practitioners and as such, we believe the subjective grading more accurately reflects real-life practice.

## Conclusions

Results from the current study demonstrate that therapeutic benefit of ACS may be related to higher levels of IL-1Ra and IGF-1. Further studies on the association between the clinical response to intra-articular ACS treatment in spontaneous traumatic OA and ACS content are warranted.

## Methods

### Study design

The study was designed as a single centre prospective cohort study, recruiting client-owned harness racehorses with a suspected low-grade joint related lameness problem via advertising in a national harness racing magazine. In order to be eligible for inclusion, horses had to be systemically healthy based on clinical examination, hematology and serum amyloid A (anti-SAA coated latex agglutination photometric immunoassay, reference range 0–20 mg/L) analysis; have no history of receiving intra-articular treatments during the last 3 months or pain-relieving medication/treatment within the last 3 days; have detectable lameness which could be abolished or significantly improved with intra-articular anesthesia as detailed below; and have no radiographic signs of moderate to marked OA defined as obvious periarticular remodeling, joint narrowing, and subchondral bone sclerosis and/or lysis. Radiographic signs of mild periarticular remodeling, defined as small and smoothly marginated new bone formation, were accepted. Signalment including age, breed and sex were recorded, and a signed consent form was obtained from owners of all horses included in the study. Horses were excluded from follow-up analyses if they received additional treatments for lameness such as intra-articular medication, extracorporeal shockwave therapy or surgical intervention during the study period.

### Clinical examination

A thorough lameness examination including detailed palpation of the extremities and back was performed in all horses by two of the authors (PMS and CTF), and abnormal findings such as joint effusion or soft tissue abnormalities were recorded. Horses were walked and trotted in hand in a straight line on a hard surface, and standardized flexion tests (60 s for the proximal forelimb, distal fore- and hind limb; and 90 s for the proximal hind limb) were performed. The examiner performing flexion tests was responsible for attributing a subjective lameness score (AAEP scale 0–5) for the initial trot-up and for each of the flexion tests and was blinded to the result of the objective analysis which was carried out by another individual (ASK). The objective analysis consisted of using a wireless inertial sensor-based lameness evaluation system,^1^ and the analysis output data (millimetres asymmetry between right and left limbs) was recorded.

Diagnostic anaesthesia using mepivacaine was performed using aseptic technique in a routine fashion. In brief, synovial fluid was aspirated to confirm intra-articular needle placement before injecting a joint-specific volume of 2% mepivacaine^2^; 10 ml was used for carpal joints (radiocarpal and intercarpal joints were injected simultaneously and hereafter referred to as the carpus) and for the tarsocrural joint as well as for each of the stifle compartments; 8 ml was used for metacarpophalangeal and metatarsophalangeal joints; whereas 4 ml was used for distal interphalangeal joints. Using routine settings, standard digital radiographic projections were acquired of all joints responding to intra-articular analgesia. In brief, this included dorso-palmar/plantar; lateromedial; dorsolateral-palmaro/plantaromedial oblique; and dorsomedial-palmaro/plantarolateral oblique projections of the carpus, tarsus and phalangeal joints; flexed dorsoproximal-dorsodistal projections centered on each of the proximal and distal row of carpal bones, respectively; and a lateromedial and caudocranial projection of the stifle.

Horses were re-evaluated (both subjectively and objectively) after 10 and 30 min. The lameness was considered localized to a specific joint when the subjective lameness grading decreased by at least 1 grade (AAEP score) within 10 min, and when the objective asymmetry score (in mm) of that limb decreased at least 50% within the same time frame. When the initial lameness was abolished or substantially improved by diagnostic analgesia, this joint was referred to as the primary problem. Secondary problems were identified by continuing the diagnostic analgesic injections in the same limb in the event of residual lameness, or in another limb (often contralateral), when horses shifted their lameness after the primary problem had been blocked. Horses with more than two identified painful joints were excluded. In the event of identifying two localized problems, both were treated with ACS, however, only data from the primary problem was used for outcome analyses.

### ACS preparation

Upon inclusion in the study, blood was drawn from a single jugular vein in an aseptic fashion through a 16G butterfly needle and ACS was prepared using commercially available kits^3^ and as per the manufacturer’s instructions. ACS containers were incubated at 37 °C for 22–24 h, prior to centrifugation (4000 rpm for 10 min) and serum collection. The resultant ACS was filtered through a 0.2 μm filter,^4^ whereupon one aliquot was immediately used for the first joint injection(s). Subsequent aliquots were stored at − 80 °C for future treatments and ACS content analyses.

### Treatment and re-evaluation

All included horses received a standardized ACS treatment course consisting of 3 intra-articular injections administered at approximately 2-week intervals; the exact treatment intervals and ACS volumes were registered per case. In general, horses received a total volume of 5 ml ACS per treatment, however, for a few horses the total extracted ACS volume was less than 15 ml, resulting in 2.5–3.5 ml being used per injection. Owners received a written standardized exercise programme recommending 48 h stall rest following each injection. Briefly, daily walking exercise for 20 min was recommended between the first and second treatment; this was increased to 30 min between the second and the third treatment, and further increased to 40 min for 2 weeks after the third injection.

Owners were encouraged to present their horses for re-evaluation (clinical examination including palpation; subjective and objective lameness evaluation including flexion tests) 2 weeks after the third injection; the exact follow-up time was recorded per case. Adverse effects were recorded for all treatments and at re-evaluation. Changes in AAEP scores from inclusion to re-evaluation were used to categorize clinical outcome; horses were considered to have responded to treatment when the subjective AAEP score was 0 on trot-up at re-evaluation, and when the flexion test scores were at least 50% reduced.

### Cytokine analyses

Equine specific commercially available solid phase sandwich ELISA kits^5^ were used for determining ACS concentration of IL-1Ra (DY2466), IL-1β (DY3340), IL-10 (DY1605) and TNFα (DY1814), whereas human-specific kits were used for TGF-β (DY240) and IGF-1 (DY291), the latter two having confirmed equine cross-reactivity [26, 27]. Pre-treatment of ACS samples prior to analysis for IGF-1 content was performed as described by Daughaday et al. [28]. For TGF-β content analysis of ACS, samples were pre-activated using Activation kit 1 (DY010). All standards and samples were assayed in duplicates and standard curves were generated for each set of samples assayed. The reported samples were diluted 1:14 for IGF-1, 1:10 for TGF- β, 1:2 for IL-1Ra, IL-1β and IL-10 and neat for TNF-α. Twofold dilutions of kit standards were set up as follows (ng/ml): 20–0,31 for IL-1Ra and IL-10, 8–0,125 for IL-1β and 2–0,031 for the remaining analytes. All assays were performed according to the manufacturer’ instructions and plates were read using a microplate reader^6^ set to 450 nm.

### Statistical analyses

Spearman’ rho correlation analyses were performed between the subjective and objective lameness evaluation methods at inclusion (trot-up, after flexion and after diagnostic anaesthesia) and at re-evaluation (trot-up and after flexion). When significantly correlated, the AAEP scores were used for further analyses. Due to non-normal distribution of clinical variables, the Wilcoxon’s rank sum test was used to determine differences between outcome groups for age; inclusion AAEP scores for trot-up and flexion; treatment interval in days; days to follow-up; and the ACS volume used.

Due to positive skewed distributions, the ACS content of IL-1Ra, IGF-1 and TGF-β were transformed using log transformation until normal distributions were confirmed by the Shapiro-Wilkes test prior to further analyses. Association between the clinical outcome (responders vs. non-responders) and age, sex, inclusion AAEP scores for trot-up and flexion, and the ACS levels of investigated cytokines and growth factors was determined using univariable logistic regression analyses. Variables associated with the outcome with a value of *P* ≤ 0.20 were included in a multivariable logistic regression model; model fit was assessed.

## Data Availability

The datasets used and analysed during the current study are available from the corresponding author upon reasonable request.

## Abbreviations

ACS: Autologous conditioned serum
OA: Osteoarthritis
IL-1Ra: Interleukin 1 receptor antagonist
IL-10: Interleukin 10
TGF- β: Transforming growth factor β
IGF-1: Insulin-like growth factor 1
PDGF: Platelet-derived growth factor
MCPJ: Metacarpophalangeal joint
TCJ: Tarsocrural joint
DIPJ: Distal interphalangeal joint
MTPJ: Metatarsophalangeal joint
AAEP: American Association of Equine Practitioners
SAA: Serum Amyloid A
TNF-α: Tumor necrosis factor α

## Footnotes

1 Equinosis Lameness Locator

2 Mepidor vet; Richter Pharma AG, Wels, Austria

3 Arthrex ABPS

4 Sterifix® 0.2 μm Luer Lock; Braun, Meslungen, Germany

5 R&D Systems Europe Ltd.; Abingdon, England, UK

6 Multiskan FC Microplate Photometer; Thermo Fisher Scientific, Massachusetts, US
